# Elevated temporal tau PET predicts faster cognitive decline in women than men: A meta‐analysis

**DOI:** 10.1002/alz.71031

**Published:** 2026-02-18

**Authors:** Annie Li, Hannah M. Klinger, Mabel Seto, Colin Birkenbihl, Michael J. Properzi, Michelle Farrell, Emma Thibault, Aaron P. Schultz, Diana L. Townsend, Madison Cuppels, Jane A. Brown, Kathryn V. Papp, Rebecca E. Amariglio, Hyun‐Sik Yang, Michael C. Donohue, Robert A. Rissman, Tobey J. Betthauser, Rebecca E. Langhough, Erin M. Jonaitis, Karly Cody, Sterling C. Johnson, Dorene M. Rentz, Keith A. Johnson, Reisa A. Sperling, Rachel F. Buckley, Gillian T. Coughlan, the A4 Study Team

**Affiliations:** ^1^ Department of Neurology Massachusetts General Hospital Harvard Medical School Boston Massachusetts USA; ^2^ Department of Neurology Brigham and Women's Hospital Boston Massachusetts USA; ^3^ Alzheimer's Therapeutic Research Institute University of Southern California San Diego California USA; ^4^ Alzheimer's Disease Research Center University of Wisconsin‐Madison Madison Wisconsin USA; ^5^ Melbourne School of Psychological Sciences University of Melbourne Melbourne Victoria Australia

**Keywords:** cognitive decline, longitudinal, preclinical Alzheimer's disease, sex differences, tau PET

## Abstract

**INTRODUCTION:**

Women show higher levels of Alzheimer's disease (AD) pathology than men, but the implications for cognitive decline remain unclear. Determining the extent to which tau burden differentially accelerates cognitive decline in men and women will provide critical insights into sex‐specific pathways of disease progression.

**METHODS:**

We leveraged tau positron emission tomography (PET), amyloid beta (Aβ) PET, apolipoprotein E (*APOE)* ε4 genotyping, and longitudinal cognitive data over approximately 8.6 (standard deviation [SD] = 3.8) years from 1007 cognitively unimpaired adults across three cohorts. Cognitive trajectories were modeled with linear mixed‐effects regression including sex × tau × time interactions, and results were synthesized using random‐effects meta‐analysis.

**RESULTS:**

Higher tau burden in medial and lateral temporal regions was associated with faster cognitive decline in women than in men.

**DISCUSSION:**

High tau burden carries a disproportionately greater cognitive cost for women, underscoring the need for sex‐specific approaches to early detection and therapeutic intervention in AD.

**Highlights:**

- A meta‐analysis across three independent cohorts shows that female cognitive advantage at low tau shifts to vulnerability at higher tau.
- Sex differences in tau‐related cognitive decline were consistent after accounting for amyloid burden.
- Sex‐specific rates of cognitive decline should be considered in clinical trial design.

## 1 BACKGROUND

While sex differences in incidence rates for Alzheimer's disease (AD) dementia are unclear,^1^ prevalence rates are undoubtedly higher for women.^2^, ^3^ Both *post mortem* findings^4^, ^5^ and positron emission tomography (PET) studies^6^, ^7^ report greater regional tau aggregation in women compared with age‐matched men, particularly in medial and lateral temporal brain areas.^6^, ^8^, ^9^, ^10^, ^11^ Sex differences in tau burden are especially evident in the context of elevated amyloid beta (Aβ)^8^, ^12^, ^13^ and apolipoprotein E (*APOE)* ε4 genotype,^14^, ^15^ but sex differences in cognitive decline as a function of tau burden are not well studied.^9^

The existing literature on sex‐specific rates of cognitive change across the AD clinical spectrum shows mixed results.^9^, ^16^, ^17^, ^18^, ^19^ This inconsistency arises in part from heterogeneity in sample characteristics, including age, education, Aβ burden, *APOE* ε4 status, and potential vascular co‐morbidities.^20^ Further, a female cognitive advantage has been reported whereby stronger performance on specific cognitive domains like verbal memory among women delays, or even underestimates, clinical detection of impairment.^21^, ^22^ Existing Aβ PET and Aβ cerebrospinal fluid (CSF) studies have also yielded conflicting results, with some reporting a significant moderating effect of sex on the association between Aβ burden and cognition over time^22^, ^23^, ^24^ and others reporting no influence.^16^, ^25^

Tau pathology is more proximal to cognitive decline than Aβ,^26^, ^27^, ^28^ but few studies have investigated the sex‐specific role of tau in predicting cognitive decline, particularly in the preclinical phase of AD. A recent single‐site study found that higher meta‐temporal tau burden predicted faster cognitive decline in women relative to men among cognitively unimpaired older adults.^9^ We substantiate and extend this research question in a large longitudinal meta‐analysis study of more than 1000 participants, incorporating over half a decade of cognitive assessment data drawn from a clinical trial and two observational studies. Our primary objective was to assess the role of sex in the association between regional tau burden and cognitive trajectories over time. To further contextualize the unique role of tau during the preclinical phase of AD, we also examined how Aβ burden and *APOE* ε4 status impacted sex‐specific rates of cognitive decline.

## 2 MATERIALS AND METHODS

### 2.1 Participants

**FIGURE 1 alz71031-fig-0001:**
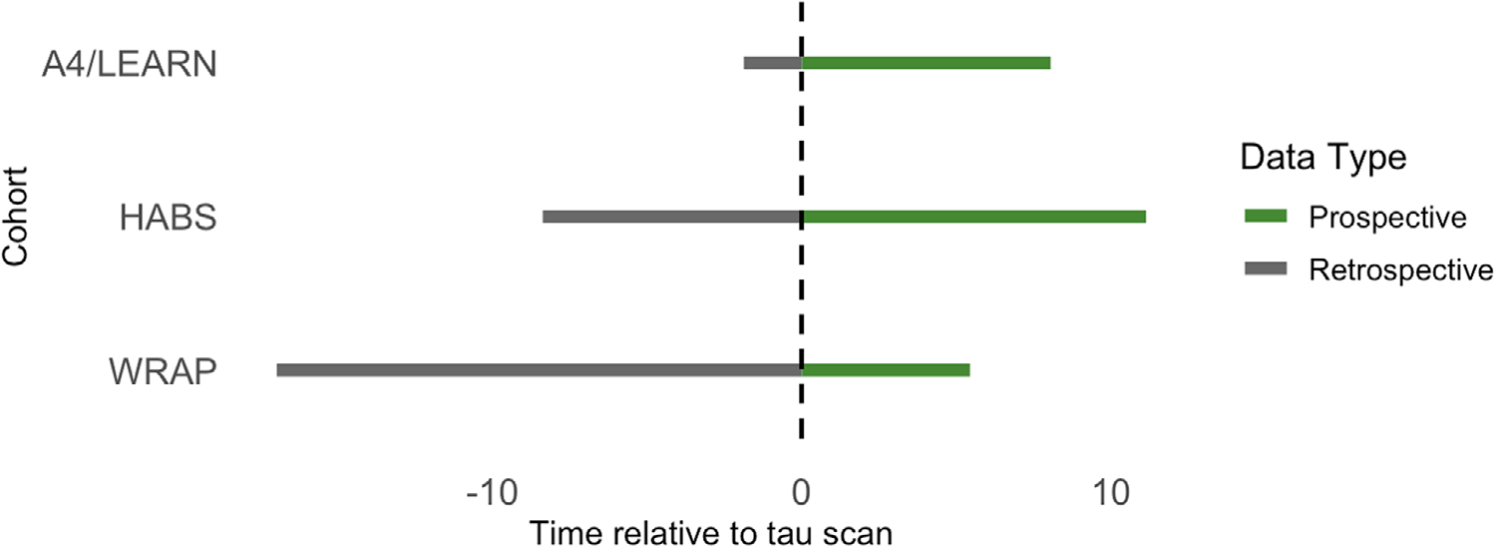
Cognitive assessment timeline span around tau PET scan by cohort. Time span of retrospective (gray) and prospective (green) cognitive assessments relative to the tau PET scan (dashed line at time = 0) across A4/LEARN, HABS, and WRAP. A4/LEARN and HABS included both retrospective and prospective assessments, whereas WRAP assessments were primarily retrospective. A4, Anti‐Amyloid Treatment in Asymptomatic Alzheimer's Study; HABS, Harvard Aging Brain Study; LEARN, Longitudinal Evaluation of Amyloid Risk and Neurodegeneration Study; PET, positron emission tomography; WRAP, Wisconsin Registry for Alzheimer's Prevention.

We analyzed data from 1007 cognitively unimpaired participants (age = 70.3 [7.5] years, range 46 to 93 years; 648 women [64%], *APOE* ε4 carrier status available for 951 participants, of whom 367 [39%] were carriers; Table 1) who were participants in the Anti‐Amyloid Treatment in Asymptomatic Alzheimer's (A4) Study and its companion Longitudinal Evaluation of Amyloid Risk and Neurodegeneration (LEARN) Study, the Harvard Aging Brain Study (HABS), and the Wisconsin Registry for Alzheimer's Prevention (WRAP) studies. Study procedures for A4/LEARN, HABS, and WRAP have been described along with their inclusion criteria.^29^, ^30^, ^31^, ^32^ In the present study, we included individuals who were cognitively unimpaired at the time of their first cognitive assessment and had both a tau PET scan and at least one cognitive assessment (Figure S1). We focused on cognitively unimpaired participants to capture disease progression during the window in which disease‐modifying therapies may offer the greatest benefit. We included participants from the placebo arm of the A4 study and the LEARN study, the latter comprising individuals who did not meet the criteria for A4 due to being Aβ‐negative at baseline, which were analyzed as a single cohort.^33^ Since placebo participants from A4 were eligible for Solanezumab treatment during the open‐label extension (OLE) period,^31^ we accounted for treatment effects by covarying for the cumulative treatment exposure at each assessment during OLE.^34^ All available cognitive assessments (*n*_Observations_ = 7927) – both preceding (*n*_Observations_ = 3102) and following the tau scan (*n*_Observations_ = 4825) – were included, with time anchored (time = 0) at the tau PET scan time point (mean [SD] = 0.22 [5.0] years, range = 17.0 to 11.1 years; Figure 1). The average cognitive follow‐up time after tau PET was 3.0 (SD = 2.1) years in A4/LEARN, 4.1 (SD = 2.8) years in HABS, and 2.0 (SD = 1.5) years in WRAP. We followed the procedures for this study under the ethical guidelines stipulated by the Mass General Brigham (MGB) Institutional Review Board. All participants provided written informed consent.

**TABLE 1 alz71031-tbl-0001:** Participant characteristics.

	A4/LEARN			HABS			WRAP			
Characteristic	Men	Women	*p* value^b^	Men	Women	*p* value^b^	Men	Women	*p* value^b^	All
*N*	98	152		114	165		147	331		1007
Baseline tau PET age (years)	72.2 (4.7)	70.9 (4.7)	**0.01**	74.3 (8.4)	72.0 (9.3)	0.07	68.5 (7.4)	67.9 (6.9)	0.30	70.3 (7.5)
Education (years)	16.9 (2.7)	15.7 (2.8)	**<0.001**	16.5 (3.0)	16.0 (2.9)	0.09	17.2 (2.8)	16.3 (2.9)	**<0.001**	16.4 (2.9)
*APOE* ε4 carriers	46 (47%)	82 (54%)	0.30	27 (24%)	48 (29%)	0.30	51 (38%)	113 (39%)	>0.90	367 (39%)
Aβ (CL; closest to tau PET)	49.7 (39.2)	51.3 (34.9)	0.60	20.8 (28.8)	24.7 (29.7)	**0.01**	15.2 (29.8)	21.4 (35.5)	**0.03**	28.6 (35.9)
PACC score (closest to tau PET)	−0.5 (2.6)	0.2 (3.0)	**0.03**	0.0 (0.7)	0.3 (0.8)	**<0.001**	−0.5 (1.2)	0.0 (1.3)	**<0.001**	−0.1 (1.7)
PACC time (years)^c^	6.1 (1.7)	5.4 (2.1)	**0.01**	8.7 (3.9)	8.9 (3.4)	>0.90	10.5 (3.9)	9.8 (3.9)	**0.03**	8.6 (3.8)
Prospective PACC time (years)^d^	3.1 (2.1)	2.9 (2.1)	**0.01**	4.3 (2.9)	4.0 (2.7)	0.21	2.1 (1.5)	2.0 (1.5)	0.40	3.3 (2.4)
Retrospective PACC time (years)^e^	−0.2 (0.1)	−0.3 (0.3)	0.40	−1.9 (1.5)	−2.0 (1.5)	0.30	−6.1 (3.9)	−6.4 (3.9)	0.20	−4.6 (4.0)
PACC timepoints	13.8 (3.6)	12.3 (4.2)	**<0.01**	8.4 (3.5)	8.8 (3.0)	0.60	5.0 (1.5)	4.7 (1.4)	**0.04**	7.9 (4.3)

Abbreviations: Aβ, β‐Aβ; A4/LEARN, anti‐Aβ treatment in asymptomatic Alzheimer's disease trial and the companion longitudinal evaluation of Aβ risk and neurodegeneration study; CL, centiloid; HABS, Harvard Aging Brain Study; PACC, preclinical Alzheimer's cognitive composite; PACC time, number of years since the earliest available tau PET scan, calculated as PACC age–tau PET age; WRAP: Wisconsin Registry for Alzheimer's Prevention.

^a^ *n* (%); mean (SD).

^b^ Pearson's chi‐squared test; Wilcoxon rank‐sum test.

^c^ PACC time refers to average PACC follow‐up time points across participants.

^d^ Prospective PACC time refers to mean PACC time for time points occurring after tau PET scan (positive values).

^e^ Retrospective PACC time refers to mean PACC time for time points occurring before tau PET scan (negative values).

### 2.2 Cognition

The current study utilized the four‐component Preclinical Alzheimer's Cognitive Composite (PACC) as the cognitive outcome.^35^ In A4/LEARN and HABS, the PACC was constructed from four tests:[Table alz71031-tbl-0001] Free Recall plus Total (sum of free and cued) score from the Free and Cued Selective Reminding Test (FCSRT), delayed paragraph recall from Logical Memory IIA, the Digit‐Symbol Substitution Test from the Wechsler Adult Intelligence Scale‐Revised, and the Mini‐Mental State Examination (MMSE). In HABS, we used the four‐test version of PACC (referred to as PACC‐96) rather than the standard HABS PACC, which includes five cognitive tests.^36^ For all cohorts, we used PACC scores as provided in the respective datasets. In A4/LEARN, the composite was calculated by summing *z*‐scores of the component tests. Alternate versions of the component tests were administered across visits to reduce practice effects. The PACC version was included as a categorical, time‐varying covariate in all models to adjust for version‐specific differences. In HABS (PACC‐96), *z*‐scores were averaged. In WRAP,^30^, ^37^ the PACC was similarly constructed from four cognitive tests; however, it used the total score across trials of the Rey Auditory Verbal Learning Test (AVLT) instead of the FCSRT, and Logical Memory II (full story recall) rather than just the delayed paragraph recall subtest (IIA). Both the AVLT and FCSRT assess list learning and memory, although the FCSRT provides controlled encoding and cued recall, whereas the AVLT relies on free recall without cueing.

**RESEARCH IN CONTEXT**

1. **Systematic review**: Women show greater tau burden than men, especially in the presence of Aβ burden. Evidence for sex differences in cognitive decline is inconsistent, and only one study to date has examined how tau burden differentially impacts rates of cognitive decline in men and women in preclinical AD.
2. **Interpretation**: Across three longitudinal cohorts, we found that women with higher temporal tau declined faster than men. Women's initial cognitive advantage at low tau diminished as burden increased, indicating that elevated tau carries a disproportionately greater cognitive cost for women. Our findings highlight the need to consider sex‐specific rates of cognitive decline in precision medicine and clinical trial design.
3. **Future directions**: Future work should identify mechanisms underlying women's greater vulnerability to tau‐related cognitive decline and examine whether similar differences occur with tau accumulation.

### 2.3 Tau PET

A4/LEARN and HABS used the [^18^F]Flortaucipir tau PET tracer, while WRAP used the [^18^F]MK‐6240 PET tracer. Acquisition parameters and preprocessing followed cohort‐specific pipelines and have been published previously.^30^, ^31^, ^38^ Briefly, mean count images were created based on mean retention from 75 to 105 min in A4/LEARN; in HABS, initially from 80 to 100 min, later extended to 75 to 105 min; and in WRAP, from 70 to 110 min. Standardized uptake value ratios (SUVRs) were computed using cerebellar gray in A4/LEARN and HABS and inferior cerebellar gray in WRAP as reference regions. Partial volume correction was not applied across all cohorts.^7^, ^13^ Tau PET imaging was introduced at different time points across studies, and the earliest available tau PET scan for each participant[Fig alz71031-fig-0001] was used for analysis. Six a priori AD‐related tau PET regions of interest (ROIs) from the medial temporal lobe and neocortex were included across studies: the amygdala, parahippocampal gyrus, and entorhinal cortex for medial temporal regions and fusiform gyrus, inferior temporal gyrus, and middle temporal gyrus for the neocortex, similar to prior work.^39^ The only exception was the WRAP study, where the entorhinal cortex was labeled as the anterior parahippocampal gyrus. In HABS and A4/LEARN, ROIs were derived using different implementations of the FreeSurfer‐based gtmseg atlas, which is based on the Desikan–Killiany parcellation but extends to include cortical, subcortical, and extra‐brain regions.^40^ In HABS, subject‐specific gtmseg atlases were generated in each participant's native space using FreeSurfer (version 6), allowing ROI definitions to be guided by individual anatomy and tissue classification. A4/LEARN used a standardized template‐space version of the gtmseg atlas; participants’ tau PET data were spatially normalized to Montreal Neurological Institute (MNI) space, and ROIs were defined in that common space. WRAP used the Harvard Oxford atlas and automated anatomical labeling atlas with additional manual segmentation.^41^ All ROIs were analyzed as bihemispheric averages. Tau PET measures were treated as a continuous variable in all analyses.

### 2.4 Aβ PET

Aβ PET scans were acquired using [^18^F]Florbetapir (FBP) in A4/LEARN and [^11^C]Pittsburgh compound B (PiB) in HABS and WRAP. Acquisition and reconstruction parameters for each study have been published previously,^42^, ^43^, ^44^ and further details can be found in Supplementary Methods. Aβ burden was analyzed as a continuous variable in Centiloid (CL) units to enhance cross‐cohort comparability. Established conversion equations were applied to transform SUVR (A4/LEARN) and distribution volume ratio (HABS and WRAP), respectively, to the CL scale.^42^, ^45^, ^46^ For all cohorts, the FBP or PiB scan closest to each participant's first tau PET scan was selected for analysis.

### 2.5 Statistical analyses

Analyses were conducted in R version 4.4.1 (The R Foundation, Vienna, Austria). Demographic characteristics were compared between men and women using Wilcoxon rank‐sum test for continuous variables and chi‐squared tests for categorical variables. We first assessed cross‐sectional differences in regional tau burden by sex using linear regression models, adjusting for tau PET age. As a sensitivity analysis, these models were additionally adjusted for Aβ burden and *APOE* ε4.

Our primary analytic approach involved random‐effects meta‐analyses to assess the extent to which the association between regional tau burden and longitudinal cognitive decline differed by sex across cohorts. To obtain estimates for the meta‐analysis, we first fitted linear mixed‐effects (LMEs) models separately within each of the three cohorts (A4/LEARN, HABS, and WRAP). These models (Model 1) evaluated the effect of regional tau SUVR on cognitive change (PACC) varied by sex over time since the tau PET scan (calculated as PACC assessment age minus tau PET scan age). Meta‐analyses were then conducted on Model 1 using the metafor package in R. We report the global fixed effect (*β*), 95% confidence interval (CI), *p* value, and total heterogeneity across cohorts (*I* ^2^).

Meta‐analysis models included both retrospective (*n* = 3102 assessments from 1006 participants) and prospective (*n* = 4825 assessments from 758 participants) cognitive data (Figure 1), corresponding to an average of 13 assessments per participant in A4/LEARN, nine in HABS, and five in WRAP (Table 1). Retrospective assessments occurred an average of 4.6 years prior to tau PET (SD = 4.0, range: 17.0 to 0 years), and prospective assessments occurred an average of 3.3 years after tau PET (SD = 2.4, range: 0 to 11.1 years). Each model included fixed effects for tau PET age × time and education × time, as well as participant‐specific random intercepts and slopes. A4/LEARN models additionally included PACC version and cumulative treatment dose as covariates.

Next, we repeated the same meta‐analysis using prospective data only to verify that the interaction persisted in this subset. We also performed floodlight analyses using the Johnson–Neyman technique^47^ to identify levels of regional tau SUVR at which the sex‐by‐time interaction on cognitive decline emerged as statistically significant (Supplementary Methods).

To assess the need for multiple comparison corrections on independent analyses, we ran a principal component analysis to determine the extent of overlap in variance across the six tau PET regions, similar to previous studies.^48^ The principal component analysis produced one factor (Table S1), suggesting that analyses involving these regions were not entirely independent of one another. Therefore, the significance threshold was set at *α* = 0.05. Nominal two‐*sided p* values are reported.

Existing evidence suggests that Aβ and *APOE* ε4 impact cognitive decline and/or clinical progression in a sex‐specific manner.^49^, ^50^ Thus, we included additional models testing sex‐specific tau associations with cognitive decline adjusting for sex interactions with Aβ and *APOE* ε4. These models included an additional three‐way interaction term for sex × Aβ‐CL × time (Model 1A) and sex × *APOE* ε4 status × time (Model 1B). Finally, we conducted exploratory analyses testing a four‐way interaction between sex, regional tau SUVR, Aβ‐CL, and time to investigate potential synergistic effects of tau and Aβ on cognitive decline (Model 2). We conducted meta‐analyses of the sex × regional tau SUVR × time interaction terms from Model 1A, Model 1B, and Model 2, using the same procedures as for Model 1. These meta‐analyses are presented first in the Results section, followed by within‐cohort results for each model.

The specific models tested were as follows:

1. Meta‐analytic models
  - Random‐effects meta‐analyses pooling standardized regression coefficients and standard errors for the sex × regional tau SUVR × time interaction term from within‐cohort models, conducted separately for each tau region.
2. Within‐cohort models
  - Model 1: PACC ~ sex × regional tau SUVR × time + tau age × time + education × time + covariates*
  - Model 1A: PACC ~ sex × regional tau SUVR × time + sex × Aβ‐CL × time + tau age × time + education × time + covariates*
  - Model 1B: PACC ~ sex × regional tau SUVR × time + sex × *APOE* ε4 × time + tau age × time + education × time + covariates*
  - Model 2: PACC ~ sex x regional tau SUVR x Aβ‐CL x time + tau age x time + education x time + covariates*

*Covariates include age at tau PET × time, years of education × time for all cohorts, and PACC version and cumulative treatment dose for A4/LEARN only. Models also included participant‐specific random intercepts and slopes.

## 3 RESULTS

### 3.1 Demographics

Participant characteristics for each cohort are summarized in Table 1. In A4/LEARN, women were 1.3 years younger (*p* = 0.01) than men. In A4/LEARN and WRAP, women had significantly fewer years of education than men (1.2 years, *p* < 0.001; 0.9 years, *p* < 0.001, respectively). Consistent with other studies,^51^ women had higher Aβ burden than men in both HABS (*p* = 0.01) and WRAP (*p* = 0.03). Additionally, in both A4/LEARN and WRAP, men had longer cognitive assessment follow‐up than women by 0.7 years (*p* = 0.01 and *p* = 0.03, respectively). Across all cohorts, women demonstrated significantly higher PACC scores than men at the visit closest to the tau scan (*p* = 0.03 in A4/LEARN, *p* < 0.001 in HABS and WRAP). No significant sex differences were observed in *APOE* ε4 status. Cohort‐level differences were notable: A4/LEARN had the highest proportion of *APOE* ε4 carriers (51%) and substantially elevated Aβ burden (mean Aβ‐CL = 51), consistent with the enrichment for Aβ‐positive individuals.

### 3.2 Main effect of sex on tau PET ROIs

Results are presented in Table 2. Women had significantly higher regional tau PET levels than men across cohorts, with differences most consistently observed in the middle and inferior temporal gyri (Figure S2). Sex differences were also seen in the entorhinal and fusiform regions in HABS. These effects attenuated in some, but not all, tau PET regions after adjusting for Aβ‐CL (Table S2).

**TABLE 2:** Sex differences in regional tau PET.

Sex						
	A4/LEARN^a^		HABS		WRAP	
ROI	*β* ^b^(95% CI)	*p* value	*β* ^c^ (95% CI)	*p* value	*β* ^d^ (95% CI)	*p* value
Amygdala	0.05 (−0.21, 0.30)	0.71	−0.04 (−0.26, 0.18)	0.73	0.05 (−0.14, 0.24)	0.59
Parahippocampal	−0.04 (−0.30, 0.21)	0.75	0.16 (−0.07, 0.39)	0.18	0.13 (−0.06, 0.32)	0.17
Entorhinal	0.18 (−0.08, 0.43)	0.18	0.26 (0.03, 0.49)	**0.03**	0.17 (−0.02, 0.36)	0.08
Fusiform	−0.07 (−0.32, 0.19)	0.61	0.30 (0.08, 0.53)	**0.01**	0.16 (−0.03, 0.35)	0.10
Inferior temporal	−0.01 (−0.27, 0.25)	0.93	0.30 (0.07, 0.52)	**0.01**	0.33 (0.14, 0.52)	**<0.01**
Middle temporal	0.26 (0.00, 0.52)	**0.05**	0.55 (0.33, 0.77)	**<0.001**	0.37 (0.18, 0.57)	**<0.001**

*Note*: Standardized regression coefficients (*β*) from linear effects models testing the main effect of sex (women vs men) on regional tau PET signal within each cohort. Sex was coded with men as the reference group, and models were adjusted for age at tau PET scan.

Abbreviations: A4/LEARN, Anti‐Amyloid Treatment in Asymptomatic Alzheimer's Study/Longitudinal Evaluation of Amyloid Risk and Neurodegeneration Study; HABS, Harvard Aging Brain Study; tau PET, tau positron emission tomography; WRAP, Wisconsin Registry for Alzheimer's Prevention.

^a^ PACC version and cumulative dose were included as covariates.

^b^ *N* = 250, df = 247.

^c^ *N* = 279, df = 276.

^d^ *N* = 478, df = 475.

### 3.3 Sex differences in cognitive decline as a function of tau: meta‐analyses

**FIGURE 2:**
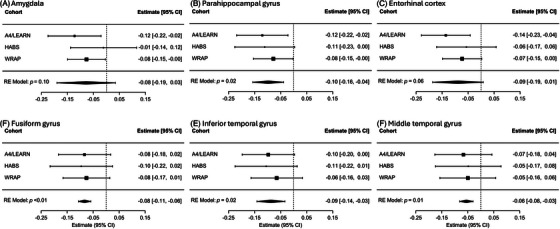
Forest plots of meta‐analytic estimates for sex × regional tau × time interaction effects on PACC trajectories. Each panel shows results from a random‐effects meta‐analysis across A4/LEARN, HABS, and WRAP cohorts for a specific ROI. Squares represent standardized regression coefficients (*β*) from each cohort, indicating the estimated difference in the effect of regional tau PET SUVR on annual change in PACC score between women and men, with men as the reference group. Horizontal lines indicate 95% confidence intervals. The diamond at the bottom of each plot represents the pooled effect from the random‐effects (RE) model. A4, Anti‐Amyloid Treatment in Asymptomatic Alzheimer's Study; HABS, Harvard Aging Brain Study; LEARN, Longitudinal Evaluation of Amyloid Risk and Neurodegeneration Study; PACC, preclinical Alzheimer's cognitive composite; ROI, region of interest; SUVR, standardized uptake value ratio; WRAP, Wisconsin Registry for Alzheimer's Prevention.

Results are presented in Table 3, with cohort‐specific and pooled estimates visualized in Figure 2. Significant interaction effects (sex × regional tau SUVR × time) were observed for the parahippocampal (*β* = −0.10, 95% CI: −0.16 to −0.04, *p* = 0.02), fusiform (*β* = −0.08, 95% CI: −0.11 to −0.06, *p* < 0.01), inferior temporal (*β* = −0.09, 95% CI: −0.14 to −0.03, *p* = 0.02), and middle temporal gyri (*β* = −0.06, 95% CI: −0.08 to −0.03, *p* = 0.01), such that women with higher medial and lateral temporal tau PET were associated with greater rates of PACC change. Effects in the amygdala (*p* = 0.10) and entorhinal cortex (*p* = 0.06) trended in the same direction but did not reach statistical significance. In the prospective sample only, significant sex effects were observed in the parahippocampal (*p* < 0.01), entorhinal (*p* = 0.04), and inferior temporal regions (*p* = 0.01) (Table S3). When accounting for the sex × Aβ‐CL × time interaction (meta‐analysis of Model 1A; Table S4), the association for higher tau in the fusiform (*p* = 0.02) and inferior temporal (*p* = 0.03) regions with faster cognitive decline in women remained significant; the sex × Aβ‑CL × time term itself was not significant in any region. After adjusting for the sex × *APOE* ε4 × time interaction (meta‐analysis of Model 1B; Table S5), the same regional tau‐related effects (parahippocampal, fusiform, inferior temporal, and middle temporal) remained statistically significant. Between‐study heterogeneity (*I* ^2^) was low across all regions (*I* ^2^ ≤ 0.5%). No region showed a significant four‐way interaction (meta‐analysis of Model 2 [sex × regional tau × Aβ‐CL × time]; Table S6).

**TABLE 3:** Meta‐analysis of sex × regional tau PET × time interaction effects on PACC trajectories.

Sex × tau × time					
ROI	*β*	95% CI	*I* ^2^ (%)	*τ* ^2^ (SE)	*p* value
Amygdala	−0.08	−0.19 to 0.03	0.30	0 (0.0017)	0.10
Parahippocampal	−0.10	−0.16 to −0.04	0.00	0 (0.0017)	**0.02**
Entorhinal	−0.09	−0.19 to 0.01	0.00	0 (0.0017)	0.06
Fusiform	−0.08	−0.11 to −0.06	0.00	0 (0.0021)	**<0.01**
Inferior temporal	−0.09	−0.14 to −0.03	0.00	0 (0.0023)	**0.02**
Middle temporal	−0.06	−0.08 to −0.03	0.00	0 (0.0027)	**0.01**

*Note*: Global fixed‐effect estimates (*β*), 95% confidence intervals (CIs), and *p* values are reported for the sex × regional tau SUVR × time interaction term pooled across A4/LEARN, HABS, and WRAP cohorts using random‐effects meta‐analysis. Coefficient estimates represent the difference in PACC change per year per unit of regional tau PET SUVR between women and men, with men as the reference group. Negative *β* values indicate stronger associations between tau burden and cognitive decline in women compared to men. *I* ^2^ reflects the percentage of total variability attributable to between‐cohort heterogeneity. *τ* ^2^ represents the estimated variance of true effect sizes across studies, with its corresponding standard error (SE).

Abbreviations: A4/LEARN, Anti‐Amyloid Treatment in Asymptomatic Alzheimer's Study/Longitudinal Evaluation of Amyloid Risk and Neurodegeneration Study; PACC, preclinical Alzheimer's cognitive composite; tau PET, tau positron emission tomography; ROI, region of interest; WRAP, Wisconsin Registry for Alzheimer's Prevention.

### 3.4 Sex differences in cognitive decline as a function of tau: within‐cohort analyses

**FIGURE 3:**
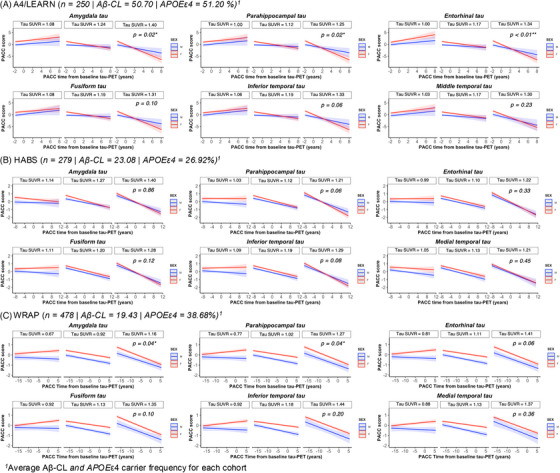
Within‐cohort analyses of sex differences in PACC trajectories as a function of regional tau PET. Three‐way linear interaction between sex and regional tau PET on PACC scores over time (*t* = 0 represents time at tau PET scan) suggests that sex moderates the association between medial temporal tau and cognitive decline in A4/LEARN and WRAP. The analysis includes both prospective and retrospective cognitive assessments. (A–C) Visualization of cohort‐specific interactions. (A) A4/LEARN (*N* = 250), (B) HABS (*N* = 279), and (C) WRAP (*N* = 478), showing relationship between sex, regional tau PET, and cognitive trajectories over time. Cognitive trajectories are faceted by tau SUVR values at mean and ± 1 standard deviation of regional tau burden within each cohort. Men were the reference group. Asterisks indicate statistical significance (*p < 0.05, **p < 0.01). A4, Anti‐Amyloid Treatment in Asymptomatic Alzheimer's Study; HABS, Harvard Aging Brain Study; LEARN, Longitudinal Evaluation of Amyloid Risk and Neurodegeneration Study; PACC, preclinical Alzheimer's cognitive composite; PET, positron emission tomography; SUVR, standardized uptake value ratio; WRAP, Wisconsin Registry for Alzheimer's Prevention.

Results are presented in Figure 3. Higher tau levels in the amygdala and parahippocampal regions were associated with steeper PACC trajectories in women compared with men (Model 1). At lower levels of tau, women consistently performed better than men on the PACC over time. This effect was observed in both A4/LEARN and WRAP (Table 4; Figure 3A,C). Sex also moderated the association between the entorhinal tau and cognitive decline in A4/LEARN, with women showing steeper slope relative to men at higher tau levels. In HABS, we did not observe a moderating effect of sex on the association between any tau region and PACC decline (Table 4; Figure 3B). Sensitivity analyses adjusting for Aβ‐CL and *APOE* ε4 status over time did not alter the findings (Supplementary Result and Tables S7,S8). Floodlight analyses identified regional and tracer‐specific tau thresholds at which sex differences in cognitive decline emerge (see Supplementary Result for results; Figure S3). Results from Model 1 using prospective‐only cognitive data (i.e., excluding retrospective data) were largely consistent with the above findings (Figure S4 and Table S9).

**TABLE 4:** Within‐cohort sex × regional tau PET × time interaction on PACC trajectories.

Sex × regional tau PET × time						
	A4/LEARN^a^		HABS		WRAP	
ROI	*β* ^b^ (95% CI)	*p* value	*β* ^c^ (95% CI)	*p* value	*β* ^d^ (95% CI)	*p* value
Amygdala	−0.12 (−0.22, −0.02)	**0.02**	−0.01 (−0.14, 0.12)	0.86	−0.08 (−0.15, −0.00)	**0.04**
Parahippocampal	−0.12 (−0.22, −0.02)	**0.02**	−0.11(−0.23, 0.00)	0.06	−0.08 (−0.15, −0.00)	**0.04**
Entorhinal	−0.14 (−0.23, −0.04)	**<0.01**	−0.06 (−0.17, 0.06)	0.33	−0.07 (−0.15, 0.00)	0.06
Fusiform	−0.08 (−0.18, 0.02)	0.10	−0.10 (−0.22, 0.02)	0.12	−0.08 (−0.17, 0.01)	0.10
Inferior temporal	−0.10 (−0.20, 0.00)	0.06	−0.11 (−0.22, 0.01)	0.08	−0.07 (−0.16, 0.03)	0.20
Middle temporal	−0.07 (−0.18, 0.04)	0.23	−0.05 (−0.17, 0.08)	0.45	−0.05 (−0.16, 0.06)	0.36

*Note*: Standardized regression coefficients from linear mixed‐effects models within each cohort, examining the interaction between sex and regional tau on cognitive decline. Men were the reference group. Models were adjusted for tau PET age and years of education over time. Negative *β* values indicate stronger associations between higher tau and faster cognitive decline in women compared to men.

Abbreviations: A4/LEARN, Anti‐Amyloid Treatment in Asymptomatic Alzheimer's Study/Longitudinal Evaluation of Amyloid Risk and Neurodegeneration Study; HABS, Harvard Aging Brain Study; PACC, preclinical Alzheimer's cognitive composite; WRAP, Wisconsin Registry for Alzheimer's Prevention.

^a^ PACC version and cumulative dose were included as covariates.

^b^ *N* = 250, df = 2960.

^c^ *N* = 279, df = 2121.

^d^ *N* = 478, df = 1818.

### 3.5 Sex differences in cognitive decline as a function of tau: within‐cohort analyses adjusting for sex‐specific amyloid effects

To assess whether sex differences in tau‐related cognitive decline were independent of interactive effects between sex and amyloid burden, we re‐estimated within‐cohort sex × regional tau × time models, including a sex × Aβ‐CL × time term (Model 1A). In A4/LEARN, significant interactions remained in the amygdala, parahippocampal gyrus, and entorhinal cortex. Consistent with the primary analyses, no significant interactions were observed in HABS. In WRAP, previously significant interactions were attenuated and no longer reached significance after adjustment. Full results are provided in Table S10. Refer to the earlier section “Sex differences in cognitive decline as a function of tau: meta‐analyses” for meta‐analytical results.

### 3.6 Sex differences in cognitive decline as a function of tau: within‐cohort analyses adjusting for sex‐specific *APOE* ε4 effects

In A4/LEARN, after controlling for sex × *APOE* ε4 interaction over time (Model 1B), regional tau in the amygdala, parahippocampal gyrus, and entorhinal cortex remained significantly associated with steeper PACC decline in women versus men. No region showed a significant sex × tau × time interaction in HABS. In WRAP, the amygdala interaction was attenuated, while significant effects remained in the parahippocampal and entorhinal regions. Full results are provided in Table S11. Refer to the earlier section “Sex differences in cognitive decline as a function of tau: meta‐analyses” for meta‐analytical results.

### 3.7 Sex differences in cognitive decline as a function of both tau and Aβ burden: within‐cohort analyses

Exploratory models tested a four‐way interaction between sex, regional tau, Aβ burden, and time (Model 2). While some significant effects emerged within individual cohorts, suggesting potential synergistic effects of tau and Aβ on cognitive decline in women these effects were not consistently observed across studies. Full results by region and cohort are presented in Table S12. Refer to the earlier section “Sex differences in cognitive decline as a function of tau: meta‐analyses” for meta‐analytical results.

## 4 DISCUSSION

This large‐scale, multicohort tau PET study of over 1000 cognitively unimpaired adults followed for up to 8.6 years provides robust evidence that sex differences in cognitive decline are associated with elevated temporal tau pathology. Across three independent cohorts, women demonstrated steeper cognitive decline than men at higher levels of medial temporal tau. Although women generally performed better than men at lower tau levels, they declined more rapidly at higher levels of tau burden, suggesting a loss of cognitive resilience associated with elevated tau pathology. The meta‐analyses provided robust support for these significant sex differences in tau‐related cognitive decline across both medial and lateral temporal regions.

Meta‐analytic models revealed consistent sex‐related effects in the parahippocampal gyrus and lateral temporal regions including the fusiform, inferior temporal, and middle temporal gyri. These findings suggest that women may be more susceptible to tau‐related cognitive decline not only in “early” medial temporal regions but also in AD‐relevant neocortical areas implicated in later stages of disease progression. The consistency of direction of effect across independent cohorts, coupled with minimal between‐study heterogeneity, supports the interpretation that these sex differences on cognitive decline reflect a replicable biological phenomenon rather than selection/recruitment bias. These results extend our previous work linking higher meta‐temporal tau burden to steeper decline in women,^9^ now demonstrated in a meta‐analysis.

Further secondary analyses revealed that women's accelerated cognitive decline in the presence of fusiform and inferior temporal tau pathology persisted after adjustment for Aβ burden or genetic risk factors. Within the same analysis, amyloid showed no sex‐dependent association with cognitive decline once the regional tau PET signal was included. Taken together, our results support the interpretation that sex differences in cognitive decline are not explained by Aβ alone^9^, ^23^, ^24^, ^49^ and may be more closely associated with tau in the temporal neocortex. Controlling for sex‐specific *APOE* ε4 effects did not alter the tau‑by‑sex association, sitting counter to other work showing that female *APOE* ε4 carriers showed greater incidence rates to AD related mild cognitive impairment and dementia.^50^ However, this is consistent with the notion that Aβ deposition is more proximally linked to cognitive decline than the genetic risk conferred by *APOE* ε4.^52^

Although the magnitude of the sex effect varied across cohorts, strongest in A4/LEARN and weakest in HABS, our meta‐analysis confirmed that the overall pattern of sex‐linked vulnerability is robust across study populations. A4/LEARN, which combines Aβ‑positive A4 participants (78 %) with Aβ‑negative LEARN participants (22 %), is a higher‐risk group for cognitive decline,^12^ whereas HABS, an observational cohort with the lowest *APOE* ε4 prevalence, exhibited only trend‑level effects. These cohort differences (driven by recruitment strategies, baseline Aβ burden, and ε4 frequency) modulate effect size but do not attenuate the consistent sex‐related signal detected in meta‐analysis. While our results implicate tau pathology as a primary driver, additional sex‐specific factors, such as post‐menopausal estrogen depletion,^53^, ^54^ heightened inflammatory responses,^55^ reduced synaptic reserve with earlier menopause,^56^ and X‐linked factors such as elevated *USP11* expression^57^, may amplify its cognitive impact.

Aligning with prior work,^8^, ^9^, ^58^ our cross‐sectional findings on sex differences in regional tauopathy show women exhibit elevated tau burden compared with men, particularly in temporal neocortical regions such as the middle and inferior temporal gyri, even after adjusting for Aβ burden. These findings were consistent across all three cohorts. Although we did not investigate longitudinal tau accumulation directly, the involvement of both early (medial temporal)‐ and later (neocortical)‐affected regions in sex‐specific rates of cognitive decline aligns with emerging longitudinal tau PET studies reporting faster accumulation of temporal and parietal tau in women.^48^, ^59^ Determining the extent to which accelerated tau accumulation drives women's steeper cognitive decline is a critical next step.

The strength of this study includes its large, multicohort design across three well‐characterized independent cohorts with long‐term follow‐up. The replication of sex differences across studies employing different tau PET tracers further supports the biological validity of these effects. However, variations in the timing of tau PET implementation across studies may have influenced the detection of sex differences at differing disease stages. Additionally, test–retest effects, particularly in cohorts with more frequent or prolonged cognitive assessments, like A4/LEARN and WRAP, may have introduced variability in estimated trajectories due to practice‐related gains in performance. Standardizing tau PET acquisition timelines or applying modeling approaches, such as adjusting for tau spread over time or staging individuals by tau propagation rather than baseline burden, could improve cross‐cohort comparability. Another limitation is that the PACC composites differed somewhat across cohorts, both in computation (sum vs average of *z*‐scores) and in specific cognitive measures (e.g., FCSRT vs AVLT; Logical Memory IIA vs II). Although these measures assess overlapping cognitive domains, minor differences in test sensitivity likely remain. Taken together, these factors introduce between‐cohort heterogeneity, but the significant pooled estimate of the meta‐analysis supports the robustness of our primary finding despite this methodological difference across cohorts. Finally, the over‐representation of highly educated, non‐Hispanic White participants in all three cohorts may limit the applicability of these findings to populations with greater racial, ethnic, and socioeconomic diversity.

In conclusion, our findings highlight that sex differences in tau‐related cognitive vulnerability span both medial temporal and lateral temporal regions. Women initially outperformed men at low tau burden, but this advantage diminished as the burden increased, resulting in greater cognitive decline. These results highlight that tau burden carries a disproportionately greater cognitive cost for women, which should be considered in clinical trial design. Collectively, our findings highlight the importance of incorporating sex‐specific tau pathology patterns into precision medicine approaches to optimize the timing and targeting of disease‐modifying therapies.

## CONFLICT OF INTEREST STATEMENT

R.A.S. has served as a consultant for AbbVie, AC Immune, Acumen, Alector, Apellis, Biohaven, Bristol Myers Squibb, Genentech, Ionis, Janssen, Oligomerix, Prothena, Roche, and Vaxxinity over the past 3 years. She has received research funding from Eisai and Eli Lilly for public–private partnership clinical trials and receives research grant funding from the NIA/NIH, GHR Foundation, and the Alzheimer's Association. Her spouse, K.A.J., reports consulting fees from Novartis, Merck, and Janssen. S.C.J. serves as a consultant to Eli Lilly, Enigma Biomedical, AlzPath, and Merck. M.C.D. serves as a consultant for Roche, and his spouse is a full‐time employee of Janssen. E.M.J. serves on a data safety monitoring board (K01 AG073587), and her spouse is employed by and has stock in Epic Systems Corporation. All other authors report no conflict of interest. Author disclosures are available in the Supporting Information.

## CONSENT STATEMENT

All subjects provided written informed consent.

## Supporting information



Supporting Information

Supporting Information
