# Supplementary material for: Elevated temporal tau PET predicts faster cognitive decline in women than men: A meta‐analysis
Source: Alzheimers Dement. 2026 Feb 18;22(2):e71031. doi: 10.1002/alz.71031 (PMC12914651; doi:10.1002/alz.71031)
Supplement: Supplementary file 1 — Supporting Information [file ALZ-22-e71031-s002.docx]

**SUPPLEMENTARY MATERIAL**

**Elevated temporal tau-PET predicts faster cognitive decline in women than men: A meta-analysis**

Annie Li^1^, Hannah M. Klinger^1^, Mabel Seto^1^, Colin Birkenbihl^1^, Michael J. Properzi^1^, Michelle Farrell^1^, Emma Thibault^1^, Aaron P. Schultz^1^, Diana L. Townsend^1^, Madison Cuppels^1^, Jane A. Brown^1^, Kathryn V. Papp^3^, Rebecca E. Amariglio^3^, Hyun-Sik Yang^1,3^, Michael C. Donohue^2^, Robert A. Rissman^2^, Tobey J. Betthauser^4^, Rebecca E. Langhough^4^, Erin Jonaitis^4^, Karly Cody^4^, Sterling C. Johnson^4^, Dorene M. Rentz^1,3^, Keith A. Johnson^1,3^, Reisa A. Sperling^1,3^, Rachel F. Buckley^1,3,5^, Gillian T. Coughlan*^1,3^ and the A4 Study Team^6^

^1^Department of Neurology, Massachusetts General Hospital, Harvard Medical School, Boston, MA, USA

^2^Alzheimer's Therapeutic Research Institute, University of Southern California, San Diego, USA

^3^Brigham and Women’s Hospital, Boston, MA, USA

^4^Alzheimer’s Disease Research Center, University of Wisconsin-Madison, WI, USA

^5^Melbourne School of Psychological Sciences, University of Melbourne, Australia

^6^A4 Study Team list available at https://www.actcinfo.org/a4-study-team-lists/

***Corresponding Author:** Gillian Coughlan, PhD. Department of Neurology, Massachusetts General Hospital, 149 13th St, Charlestown, MA, 02129 (GCOUGHLAN@mgh.harvard.edu)

Table of Contents

[Supplementary Methods 4](#_Toc211523657)

[Aβ Positron Emission Tomography (PET) Preprocessing 4](#_Toc211523658)

[Floodlight analysis 4](#_Toc211523659)

[Supplementary Results 4](#_Toc211523660)

[Within-cohort sensitivity analyses for sex differences in cognitive decline as a function of regional tau-PET (Model 1) 4](#_Toc211523661)

[Floodlight analyses for sex differences in cognitive decline as a function of regional tau-PET 4](#_Toc211523662)

[Supplementary Tables 5](#_Toc211523663)

[Supplementary Table 1: Results from the Principal Component Analysis (PCA) 5](#_Toc211523664)

[Supplementary Table 2: Sex differences in regional tau-PET, adjusting for Aβ-CL 5](#_Toc211523665)

[Supplementary Table 3. Meta-analysis of sex x regional tau-PET x time on PACC trajectories (Model 1 prospective PACC) 6](#_Toc211523666)

[Supplementary Table 4. Meta-analysis of sex x regional tau-PET x time on PACC trajectories, adjusting for sex x Aβ-CL x time (Model 1A) 6](#_Toc211523667)

[Supplementary Table 5. Meta-analysis of sex x regional tau-PET x time on PACC trajectories, adjusting for sex x *APOE*ε4 x time (Model 1B) 6](#_Toc211523668)

[Supplementary Table 6. Meta-analysis of sex x regional tau-PET x Aβ-CL x time on PACC trajectories (Model 2) 6](#_Toc211523669)

[Supplementary Table 7: Standardized regression coefficients for interactions between sex, regional tau-PET, and time on PACC trajectories, adjusting for Aβ-CL over time 7](#_Toc211523670)

[Supplementary Table 8: Standardized regression coefficients for interactions between sex, regional tau-PET, and time on PACC trajectories, adjusting for *APOE*ε4 over time (Within-cohort sensitivity analysis model 1) 7](#_Toc211523671)

[Supplementary Table 9: Standardized regression coefficients for interactions between sex, regional tau-PET, and time on prospective PACC trajectories (Within-cohort sensitivity analysis model 1) 8](#_Toc211523672)

[Supplementary Table 10: Standardized regression coefficients for interactions between sex, regional tau-PET, and time on PACC trajectories, adjusting for interactions between sex, Aβ-CL, and time (Within-cohort analysis model 1A) 8](#_Toc211523673)

[Supplementary Table 11: Standardized regression coefficients for interactions between sex, regional tau-PET, and time on PACC trajectories, adjusting for interactions between sex, *APOE*ε4, and time (Within-cohort analysis model 1B) 8](#_Toc211523674)

[Supplementary Table 12. Standardized regression coefficients for interactions between sex, regional tau-PET, Aβ-CL, and time on PACC trajectories (Within-cohort analysis model 2) 9](#_Toc211523675)

[Supplementary Figures 10](#_Toc211523676)

[Supplementary Figure 1: Participant inclusion flowcharts for A4/LEARN, HABS, and WRAP cohorts 10](#_Toc211523677)

[Supplementary Figure 2: Main effect of sex on baseline tau-PET 11](#_Toc211523678)

[Supplementary Figure 3: Floodlight thresholds for significant sex × regional tau-PET × time interaction (Model 1) 12](#_Toc211523679)

[Supplementary Figure 4: Change in prospective PACC trajectories in women and men over time as a function of tau 13](#_Toc211523680)

# **Supplementary Methods**

## **Aβ Positron Emission Tomography (PET) Preprocessing**

In brief, mean count images were generated 50–70 minutes post-injection in A4/LEARN, with measurements expressed as SUVRs normalized to a whole-cerebellum reference region. In HABS, mean count images were generated 40–60 minutes post-injection, whereas WRAP utilized images derived from a 70-minute dynamic acquisition. For both HABS and WRAP, measurements were expressed as distribution volume ratios (DVRs) using cerebellar grey as the reference region.

## **Floodlight analysis**

We extracted individual PACC slopes from a linear mixed-effects model regressing PACC on time, while accounting for participant-specific random intercepts and slopes. These slopes were then used as the dependent variable in a linear model testing the interaction between sex and baseline tau to determine the tau threshold required to observe a significant association between sex and cognitive decline.

# **Supplementary Results**

**Within-cohort sensitivity analyses for sex differences in cognitive decline as a function of regional tau-PET (Model 1)**

We conducted sensitivity analyses adjusting for baseline Aβ-CL (Supplementary Table 7) and *APOE*ε4 status (Supplementary Table 8) over time. Neither adjustment attenuated the primary interaction effects observed in the original analysis across cohorts. However, in A4/LEARN, after adjusting for baseline Aβ-CL over time, sex x regional tau x time interaction on PACC trajectories became significant in the fusiform (β = -0.11, 95% CI: -0.21 to -0.01, P = 0.031) and inferior temporal gyri (β = -0.12, 95% CI: -0.22 to -0.02, P = 0.019). In WRAP, after adjusting for baseline Aβ-CL over time, higher entorhinal tau (β = -0.09, 95% CI: -0.17 to -0.00, P = 0.041) became significantly associated with PACC decline in women compared to men. Further adjustment for *APOE*ε4 status over time revealed additional significant associations in the entorhinal (β = -0.10, 95% CI: -0.17 to -0.02, P = 0.015), fusiform (β = -0.10, 95% CI: -0.19 to -0.01, P = 0.037), and inferior temporal regions (β = -0.10, 95% CI: -0.20 to -0.00, P = 0.040). The direction of effects was consistent with the results from the primary models, suggesting that the moderating effect of sex on the association between tau and cognitive decline extends to other tau regions when additional biological AD risk factors are adjusted.

We repeated the interaction analysis using only prospective PACC data relative to the tau-PET scan (Supplementary Fig. 3). In A4/LEARN, higher baseline tau in the amygdala (β = -0.12, 95% CI: -0.22 to -0.02, p = 0.02), parahippocampal (β = -0.11, 95% CI: -0.21 to -0.01, p = 0.02), and entorhinal cortex (β = -0.13, 95% CI: -0.23 to -0.04, p < 0.01) remained significantly associated with faster rate of PACC decline in women compared to men. In WRAP, the previously significant associations in the amygdala and parahippocampal regions from Model 1 were attenuated, and tau in the fusiform gyrus emerged as a significant predictor (β = -0.24, 95% CI: -0.46 to -0.01, p = 0.04). No significant interactions were observed in HABS (Supplementary Table 9).

## **Floodlight analyses for sex differences in cognitive decline as a function of regional tau-PET**

Given the observed interactions between sex, tau burden, and cognitive decline, we conducted floodlight analyses to explore the range of tau levels at which the association between sex and rate of cognitive decline (PACC slope) reached statistical significance.

Floodlight analysis (Supplementary Fig. 4) in A4/LEARN revealed that sex differences in cognitive decline were only significant at higher tau levels—specifically, between the 80th and 99th percentile for amygdala tau, and above the 71st and 85th percentile for parahippocampal and entorhinal tau, respectively. Notably, women generally exhibited better cognitive performance at lower tau levels. A sex difference in slopes that favored men was significant only for tau levels exceeding the above thresholds. In WRAP, no significant threshold was identified for the amygdala, but a biphasic pattern was observed in the parahippocampal region, where sex differences were significant both below the 6th percentile (low tau) and above the 82nd percentile (high tau). This suggests that women performed better than men at low tau levels but declined more rapidly at higher tau levels. Tau levels were expressed as percentiles to account for differences in radiotracer and dynamic range across cohorts.

# **Supplementary Tables**

## **Supplementary Table 1: Results from the Principal Component Analysis (PCA)**


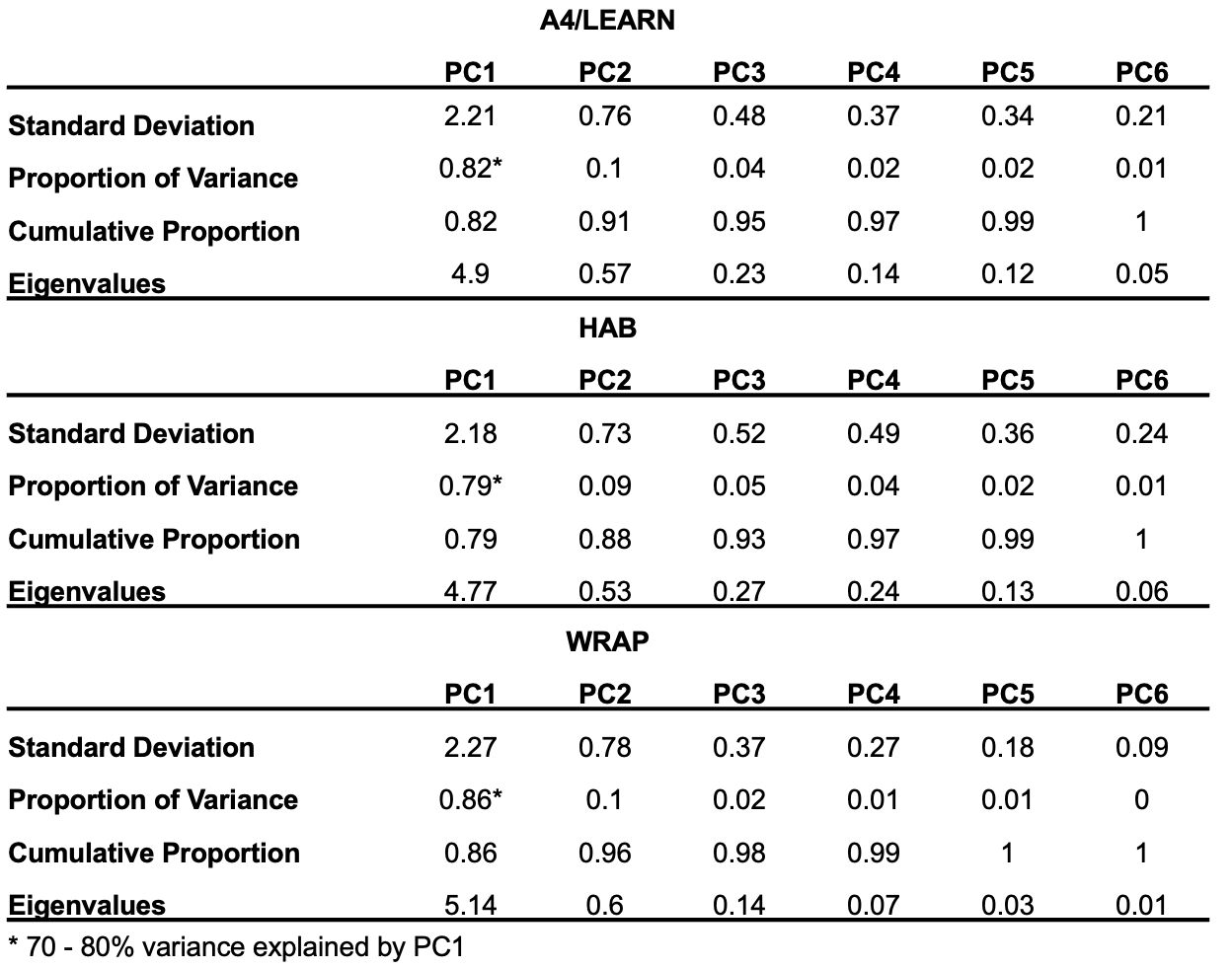


**Supplementary Table 2: Sex differences in regional tau-PET, adjusting for Aβ-CL**

| Sex | | | | | | | | | |
| --- | --- | --- | --- | --- | --- | --- | --- | --- | --- |
|  | A4/LEARN | | | HABS | | | WRAP | | |
| ROI | β^1^ | 95% CI | p-value | β^2^ | 95% CI | p-value | β^3^ | 95% CI | p-value |
| Amygdala | 0.01 | -0.23 – 0.24 | 0.96 | -0.11 | -0.32 – 0.10 | 0.32 | -0.05 | -0.20 – 0.10 | 0.50 |
| Parahippocampal | -0.08 | -0.32 – 0.16 | 0.50 | 0.08 | -0.14 – 0.30 | 0.48 | 0.02 | -0.13 – 0.18 | 0.78 |
| Entorhinal | 0.13 | -0.10 – 0.36 | 0.28 | 0.19 | -0.02 – 0.40 | 0.08 | 0.06 | -0.09 – 0.21 | 0.45 |
| Fusiform | -0.11 | -0.34 – 0.13 | 0.38 | 0.25 | 0.04 – 0.46 | **0.02** | 0.06 | -0.11 – 0.23 | 0.50 |
| Inferior temporal | -0.05 | -0.29 – 0.19 | 0.67 | 0.24 | 0.03 – 0.45 | **0.03** | 0.23 | 0.06 – 0.40 | **<0.01** |
| Middle temporal | 0.22 | -0.02 – 0.46 | 0.07 | 0.48 | 0.27 – 0.70 | **<0.001** | 0.28 | 0.11 – 0.46 | **<0.01** |

^1^N = 250, df = 246; ^2^N = 271, df = 267; ^3^N = 444, df = 440;

## **Supplementary Table 3. Meta-analysis of sex x regional tau-PET x time on PACC trajectories (Model 1 prospective PACC)**

| Sex x tau x time | | | | | | |
| --- | --- | --- | --- | --- | --- | --- |
| ROI | β | 95% CI | I^2^, % | τ^2^ (SE) | p-value |  |
| Amygdala | -0.08 | -0.23 – 0.06 | 0.16 | 0.0000 (0.0026) | 0.12 |  |
| Parahippocampal | -0.11 | -0.14 – -0.08 | 0.00 | 0.0000 (0.0027) | **<0.01** |  |
| Entorhinal | -0.11 | -0.21 – -0.01 | 0.00 | 0.0000 (0.0025) | **0.04** |  |
| Fusiform | -0.10 | -0.25 – 0.05 | 0.08 | 0.0000 (0.0030) | 0.10 |  |
| Inferior temporal | -0.10 | -0.14 – -0.07 | 0.00 | 0.0000 (0.0030) | **0.01** |  |
| Middle temporal | -0.07 | -0.19 – 0.05 | 0.00 | 0.0000 (0.0034) | 0.14 |  |

I² reflects the percentage of total variability attributable to between-cohort heterogeneity. τ² represents the estimated variance of true effect sizes across studies, with its corresponding standard error (SE).

## **Supplementary Table 4. Meta-analysis of sex x regional tau-PET x time on PACC trajectories, adjusting for sex x Aβ-CL x time (Model 1A)**

| Sex x tau x time | | | | | | |
| --- | --- | --- | --- | --- | --- | --- |
| ROI | β | 95% CI | I^2^, % | τ^2^ (SE) | p-value |  |
| Amygdala | -0.04 | -0.27 – 0.18 | 33.06 | 0.0016 (0.0039) | 0.48 |  |
| Parahippocampal | -0.08 | -0.20 – 0.04 | 0.00 | 0.0000 (0.0025) | 0.11 |  |
| Entorhinal | -0.06 | -0.17 – 0.15 | 29.61 | 0.0013 (0.0036) | 0.34 |  |
| Fusiform | -0.07 | -0.12 – 0.02 | 0.00 | 0.0000 (0.0026) | **0.02** |  |
| Inferior temporal | -0.07 | -0.13 – -0.01 | 0.00 | 0.0000 (0.0027) | **0.03** |  |
| Middle temporal | -0.03 | -0.13 – 0.06 | 0.00 | 0.0000 (0.0030) | 0.28 |  |

I² reflects the percentage of total variability attributable to between-cohort heterogeneity. τ² represents the estimated variance of true effect sizes across studies, with its corresponding standard error (SE).

## **Supplementary Table 5. Meta-analysis of sex x regional tau-PET x time on PACC trajectories, adjusting for sex x *APOE*ε4 x time (Model 1B)**

| Sex x tau x time | | | | | | |
| --- | --- | --- | --- | --- | --- | --- |
| ROI | β | 95% CI | I^2^, % | τ^2^ (SE) | p-value |  |
| Amygdala | -0.08 | -0.22 – 0.06 | 0.06 | 0 (0.0018) | 0.14 |  |
| Parahippocampal | -0.10 | -0.16 – -0.04 | 0.00 | 0 (0.0019) | **0.02** |  |
| Entorhinal | -0.10 | -0.21 – 0.02 | 0.00 | 0 (0.0018) | 0.07 |  |
| Fusiform | -0.09 | -0.10 – -0.08 | 0.00 | 0 (0.0022) | **<0.001** |  |
| Inferior temporal | -0.10 | -0.11 – -0.09 | 0.00 | 0 (0.0023) | **0.001** |  |
| Middle temporal | -0.08 | -0.12 – -0.03 | 0.00 | 0 (0.0026) | **0.02** |  |

I² reflects the percentage of total variability attributable to between-cohort heterogeneity. τ² represents the estimated variance of true effect sizes across studies, with its corresponding standard error (SE).

## **Supplementary Table 6. Meta-analysis of sex x regional tau-PET x Aβ-CL x time on PACC trajectories (Model 2)**

| Sex x tau x Aβ-CL x time | | | | | | |
| --- | --- | --- | --- | --- | --- | --- |
| ROI | β | 95% CI | I^2^, % | τ^2^ (SE) | p-value |  |
| Amygdala | -0.00 | -0.23 – 0.23 | 59.32 | 0.0029 (0.0040) | 0.95 |  |
| Parahippocampal | 0.01 | -0.14 – 0.15 | 0.00 | 0.0000 (0.0013) | 0.85 |  |
| Entorhinal | 0.01 | -0.38 – 0.39 | 88.08 | 0.0135 (0.0126) | 0.94 |  |
| Fusiform | 0.04 | -0.07 – 0.14 | 0.00 | 0.0000 (0.0014) | 0.28 |  |
| Inferior temporal | 0.04 | -0.14 – 0.23 | 39.04 | 0.0013 (0.0026) | 0.40 |  |
| Middle temporal | 0.03 | -0.07 – 0.14 | 0.00 | 0.0000 (0.0016) | 0.30 |  |

I² reflects the percentage of total variability attributable to between-cohort heterogeneity. τ² represents the estimated variance of true effect sizes across studies, with its corresponding standard error (SE).

**Supplementary Table 7: Standardized regression coefficients for interactions between sex, regional tau-PET, and time on PACC trajectories, adjusting for Aβ-CL over time** **(Within-cohort sensitivity analysis model 1)**

| Sex x tau x time | | | | | | | | | |
| --- | --- | --- | --- | --- | --- | --- | --- | --- | --- |
|  | A4/LEARN^1^ | | | HABS | | | WRAP | | |
| ROI | β^2^ | 95% CI | p-value | β^3^ | 95% CI | p-value | β^4^ | 95% CI | p-value |
| Amygdala | -0.14 | -0.23 – -0.04 | **0.01** | 0.00 | -0.12 – 0.12 | 0.99 | -0.08 | -0.16 – -0.01 | **0.04** |
| Parahippocampal | -0.14 | -0.24 – -0.05 | **<0.01** | -0.07 | -0.18 – 0.04 | 0.22 | -0.09 | -0.17 – -0.01 | **0.03** |
| Entorhinal | -0.14 | -0.23 – -0.05 | **<0.01** | -0.02 | -0.13 – 0.09 | 0.68 | -0.09 | -0.17 – -0.00 | **0.04** |
| Fusiform | -0.11 | -0.21 – -0.01 | **0.03** | -0.09 | -0.20 – 0.03 | 0.14 | -0.09 | -0.18 – 0.01 | 0.07 |
| Inferior temporal | -0.12 | -0.22 – -0.02 | **0.02** | -0.09 | -0.21 – 0.02 | 0.11 | -0.07 | -0.17 – 0.03 | 0.18 |
| Middle temporal | -0.09 | -0.20 – 0.01 | 0.10 | -0.06 | -0.17 – 0.06 | 0.36 | -0.05 | -0.16 – 0.07 | 0.43 |

^1^PACC version and cumulative dose were included as covariates; ^2^N = 250, df = 2959; ^3^N = 271, df = 2074; ^4^N = 444, df = 1687;

**Supplementary Table 8: Standardized regression coefficients for interactions between sex, regional tau-PET, and time on PACC trajectories, adjusting for *APOE*ε4 over time (Within-cohort sensitivity analysis model 1)**

| Sex x tau x time | | | | | | | | | |
| --- | --- | --- | --- | --- | --- | --- | --- | --- | --- |
|  | A4/LEARN^1^ | | | HABS | | | WRAP | | |
| ROI | β^2^ | 95% CI | p-value | β^3^ | 95% CI | p-value | β^4^ | 95% CI | p-value |
| Amygdala | -0.13 | -0.23 – -0.02 | **0.02** | 0 | -0.13 – 0.12 | 0.97 | -0.09 | -0.16 – -0.02 | **0.02** |
| Parahippocampal | -0.12 | -0.22 – -0.03 | **0.01** | -0.11 | -0.22 – 0.01 | 0.07 | -0.1 | -0.17 – -0.02 | **0.02** |
| Entorhinal | -0.14 | -0.23 – -0.04 | **0.01** | -0.04 | -0.16 – 0.07 | 0.45 | -0.1 | -0.17 – -0.02 | **0.02** |
| Fusiform | -0.08 | -0.18 – 0.02 | 0.10 | -0.09 | -0.21 – 0.03 | 0.13 | -0.1 | -0.19 – -0.01 | **0.04** |
| Inferior temporal | -0.1 | -0.20 – 0.00 | 0.06 | -0.1 | -0.22 – 0.02 | 0.09 | -0.1 | -0.20 – -0.00 | **0.04** |
| Middle temporal | -0.07 | -0.18 – 0.04 | 0.23 | -0.06 | -0.18 – 0.06 | 0.35 | -0.1 | -0.20 – 0.00 | 0.06 |

^1^PACC version and cumulative dose were included as covariates; ^2^N = 250, df = 2959; ^3^N = 277, df = 2101; ^4^N = 424, df = 1755;

## **Supplementary Table 9: Standardized regression coefficients for interactions between sex, regional tau-PET, and time on prospective PACC trajectories (Within-cohort sensitivity analysis model 1)**

| Sex tau x time | | | | | | | | | |
| --- | --- | --- | --- | --- | --- | --- | --- | --- | --- |
|  | A4/LEARN^1^ | | | HABS | | | WRAP | | |
| ROI | β^2^ | 95% CI | p-value | β^3^ | 95% CI | p-value | β^4^ | 95% CI | p-value |
| Amygdala | -0.12 | -0.22 – -0.02 | **0.02** | 0.00 | -0.14 – 0.13 | 0.97 | -0.10 | -0.21 – 0.01 | 0.08 |
| Parahippocampal | -0.11 | -0.21 – -0.01 | **0.02** | -0.1 | -0.22 – 0.02 | 0.10 | -0.13 | -0.26 – 0.01 | 0.06 |
| Entorhinal | -0.13 | -0.23 – -0.04 | **<0.01** | -0.06 | -0.17 – 0.06 | 0.32 | -0.13 | -0.26 – 0.01 | 0.06 |
| Fusiform | -0.08 | -0.18 – 0.02 | 0.14 | -0.09 | -0.21 – 0.03 | 0.14 | -0.24 | -0.46 – -0.01 | **0.04** |
| Inferior temporal | -0.10 | -0.20 – 0.00 | 0.05 | -0.1 | -0.22 – 0.02 | 0.11 | -0.14 | -0.42 – 0.14 | 0.31 |
| Middle temporal | -0.07 | -0.18 – 0.04 | 0.20 | -0.03 | -0.16 – 0.09 | 0.60 | -0.17 | -0.42 – 0.07 | 0.16 |

^1^PACC version and cumulative dose were included as covariates; ^2^N = 250, df = 2960; ^3^N = 271, df = 1292; ^4^N = 237, df = 52

## **Supplementary Table 10: Standardized regression coefficients for interactions between sex, regional tau-PET, and time on PACC trajectories, adjusting for interactions between sex, Aβ-CL, and time (Within-cohort analysis model 1A)**

| Sex x tau x time | | | | | | | | | |
| --- | --- | --- | --- | --- | --- | --- | --- | --- | --- |
|  | A4/LEARN^1^ | | | HABS | | | WRAP | | |
| ROI | β^2^ | 95% CI | p-value | β^3^ | 95% CI | p-value | β^4^ | 95% CI | p-value |
| Amygdala | -0.12 | -0.23 – -0.02 | **0.03** | 0.06 | -0.07 – 0.19 | 0.35 | -0.05 | -0.15 – 0.05 | 0.34 |
| Parahippocampal | -0.12 | -0.22 – -0.01 | **0.03** | -0.02 | -0.14 – 0.10 | 0.78 | -0.08 | -0.18 – 0.03 | 0.15 |
| Entorhinal | -0.14 | -0.24 – -0.03 | **0.01** | 0.04 | -0.09 – 0.16 | 0.56 | -0.06 | -0.16 – 0.03 | 0.26 |
| Fusiform | -0.08 | -0.18 – 0.03 | 0.17 | -0.05 | -0.17 – 0.08 | 0.48 | -0.09 | -0.19 – 0.02 | 0.11 |
| Inferior temporal | -0.09 | -0.20 – 0.01 | 0.09 | -0.05 | -0.18– 0.08 | 0.44 | -0.06 | -0.18 – 0.05 | 0.27 |
| Middle temporal | -0.07 | -0.18 – 0.05 | 0.25 | 0.01 | -0.12 – 0.14 | 0.87 | -0.03 | -0.15 – 0.08 | 0.58 |

^1^PACC version and cumulative dose were included as covariates; ^2^N = 250, df = 2958; ^3^N = 271, df = 2073; ^4^N = 444, df = 1686

## **Supplementary Table 11: Standardized regression coefficients for interactions between sex, regional tau-PET, and time on PACC trajectories, adjusting for interactions between sex, *APOE*ε4, and time (Within-cohort analysis model 1B)**

| Sex x tau x time | | | | | | | | | |
| --- | --- | --- | --- | --- | --- | --- | --- | --- | --- |
|  | A4/LEARN^1^ | | | HABS | | | WRAP | | |
| ROI | β^2^ | 95% CI | p-value | β^3^ | 95% CI | p-value | β^4^ | 95% CI | p-value |
| Amygdala | -0.14 | -0.24 – -0.03 | **0.01** | -0.00 | -0.13 – 0.12 | 0.97 | -0.07 | -0.15 – 0.00 | 0.06 |
| Parahippocampal | -0.13 | -0.23 – -0.03 | **0.01** | -0.11 | -0.23 – 0.01 | 0.07 | -0.08 | -0.16 – 0.00 | **0.05** |
| Entorhinal | -0.15 | -0.25 – -0.05 | **<0.01** | -0.05 | -0.17 – 0.07 | 0.40 | -0.08 | -0.16 – -0.00 | **0.05** |
| Fusiform | -0.09 | -0.19 – 0.01 | 0.09 | -0.09 | -0.21 – 0.32 | 0.13 | -0.09 | -0.18 – 0.00 | 0.06 |
| Inferior temporal | -0.10 | -0.21 – 0.00 | **0.05** | -0.10 | -0.22 – 0.02 | 0.09 | -0.09 | -0.19 – 0.00 | 0.06 |
| Middle temporal | -0.07 | -0.18 – 0.04 | 0.20 | -0.06 | -0.18 – 0.07 | 0.38 | -0.09 | -0.20 – 0.01 | 0.07 |

^1^PACC version and cumulative dose were included as covariates; ^2^N = 250, df = 2960; ^3^N = 271, df = 2075; ^4^N = 444, df = 1687

## **Supplementary Table 12. Standardized regression coefficients for interactions between sex, regional tau-PET, Aβ-CL, and time on PACC trajectories (Within-cohort analysis model 2)**

| Sex x tau x Aβ-CL x time | | | | | | | | | |
| --- | --- | --- | --- | --- | --- | --- | --- | --- | --- |
|  | A4/LEARN^1^ | | | HABS | | | WRAP | | |
| ROI | β^2^ | 95% CI | p-value | β^3^ | 95% CI | p-value | β^4^ | 95% CI | p-value |
| Amygdala | -0.10 | -0.19 – -0.01 | **0.04** | 0.10 | -0.01 – 0.22 | 0.09 | 0.00 | -0.06 – 0.07 | 0.92 |
| Parahippocampal | -0.05 | -0.15 – 0.05 | 0.33 | 0.09 | -0.01 – 0.19 | 0.08 | 0.00 | -0.07 – 0.06 | 0.94 |
| Entorhinal | -0.15 | -0.23 – -0.06 | **<0.01** | 0.17 | 0.07 – 0.27 | **<0.01** | 0.00 | -0.06 – 0.07 | 0.90 |
| Fusiform | -0.01 | -0.10 – 0.09 | 0.87 | 0.09 | -0.01 – 0.20 | 0.08 | 0.03 | -0.03 – 0.10 | 0.33 |
| Inferior temporal | -0.01 | -0.11 – 0.09 | 0.85 | 0.14 | 0.04 – 0.23 | **<0.01** | 0.02 | -0.05 – 0.09 | 0.59 |
| Middle temporal | 0.01 | -0.09 – 0.10 | 0.90 | 0.09 | -0.01 – 0.18 | 0.06 | 0.02 | -0.06 – 0.09 | 0.70 |

^1^PACC version and cumulative dose were included as covariates; ^2^N = 250, df = 2956; ^3^N = 271, df = 2071; ^4^N = 444, df = 1684;

# **Supplementary Figures**

**Supplementary Figure 1: Participant inclusion flowcharts for A4/LEARN, HABS, and WRAP cohorts**
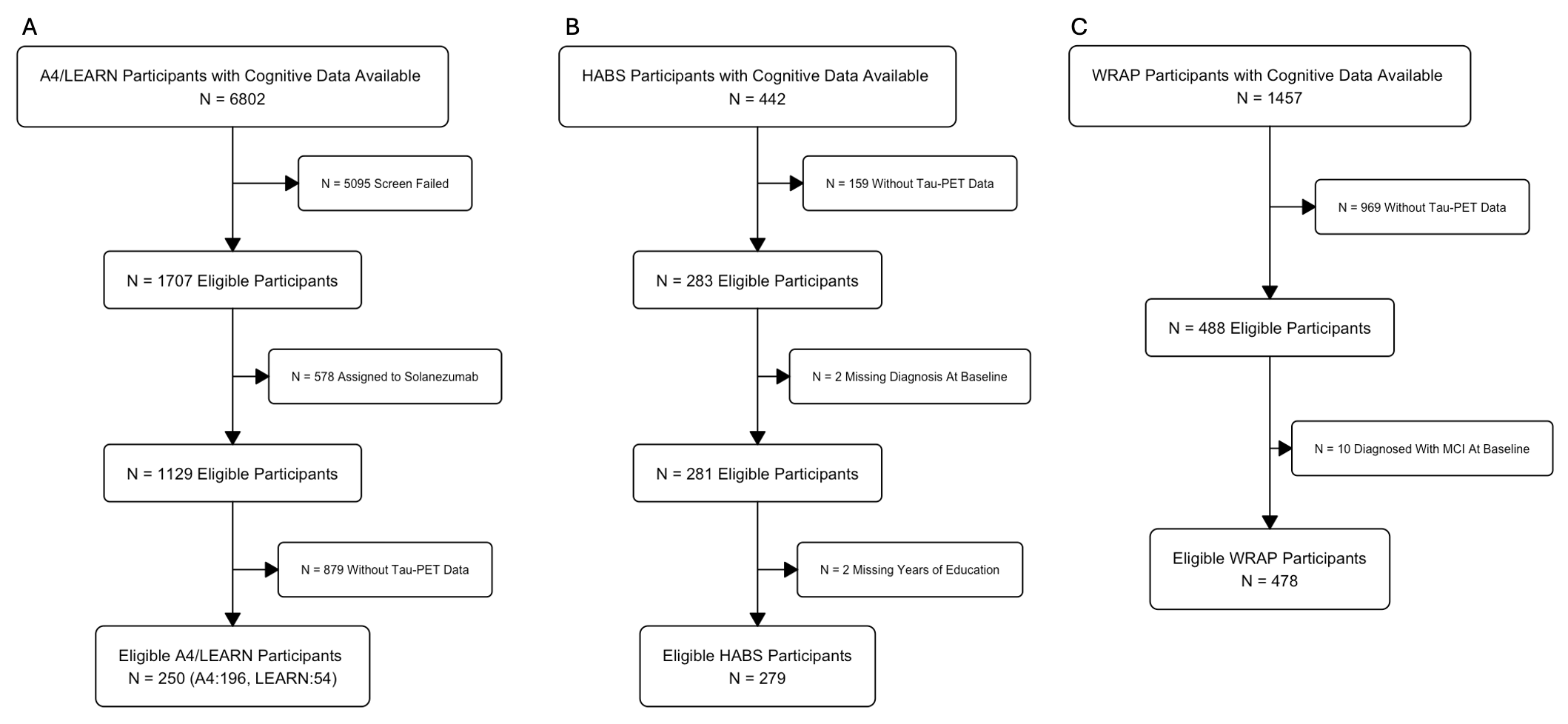


Flow diagrams (Panels A–C) illustrate cohort-specific inclusion criteria applied to derive the analytic sample for the present study. Participants with available cognitive data were sequentially filtered based on cohort-specific eligibility criteria, including availability of tau-PET imaging, baseline diagnosis, treatment arm (for A4/LEARN), and key demographic variables (e.g., years of education). Final boxes indicate the number of participants included in the analytic sample for each cohort.

## **Supplementary Figure 2: Main effect of sex on baseline tau-PET**


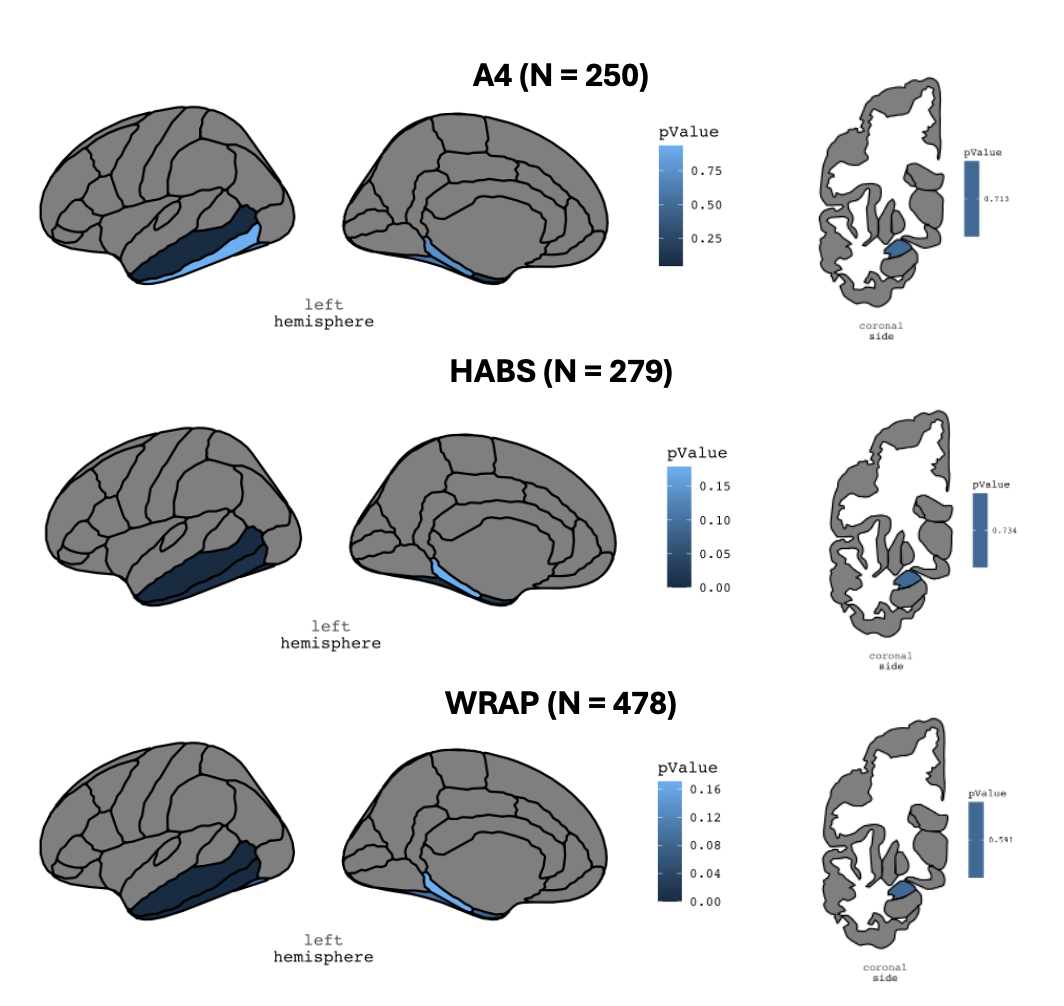


Cortical surface maps illustrating the main effect of sex on baseline tau-PET, adjusted for tau age. Across all three cohorts, women showed significantly greater tau burden than men in the middle temporal gyri. Additional significant sex effects were observed in neocortical regions in HABS and WRAP, including the inferior temporal and fusiform gyri. In HABS, the entorhinal cortex also showed a significant effect. For visualization, we focused on the left hemisphere and included five cortical regions using the Desikan-Killiany atlas. The amygdala, a subcortical structure, was visualized separately using the aseg atlas.

## **Supplementary Figure 3: Floodlight thresholds for significant sex × regional tau-PET × time interaction (Model 1)**


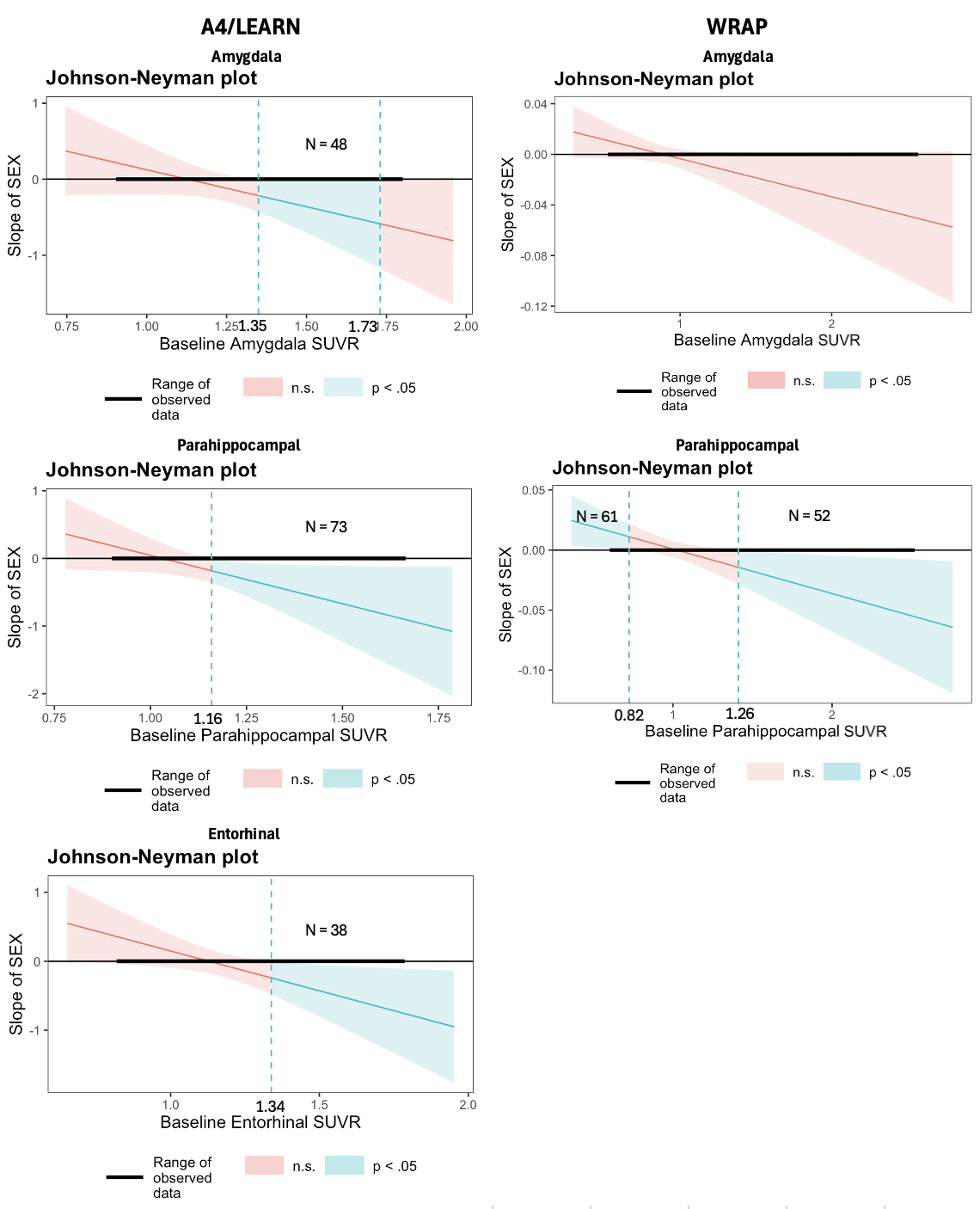


Johnson-Neyman plots for tau regions from primary models with significant three-way interactions, showing the tau SUVR threshold at which sex differences in cognitive decline emerge. The Y-axis represents the estimated effect of sex (with men as the reference group) on the slope of PACC with respect to time. The green shaded area indicates the range of tau SUVR values where the association between sex and PACC rate of change is statistically significant (*p* < .05), with a negative slope indicating faster decline in women compared to men.

## **Supplementary Figure 4: Change in prospective PACC trajectories in women and men over time as a function of tau**


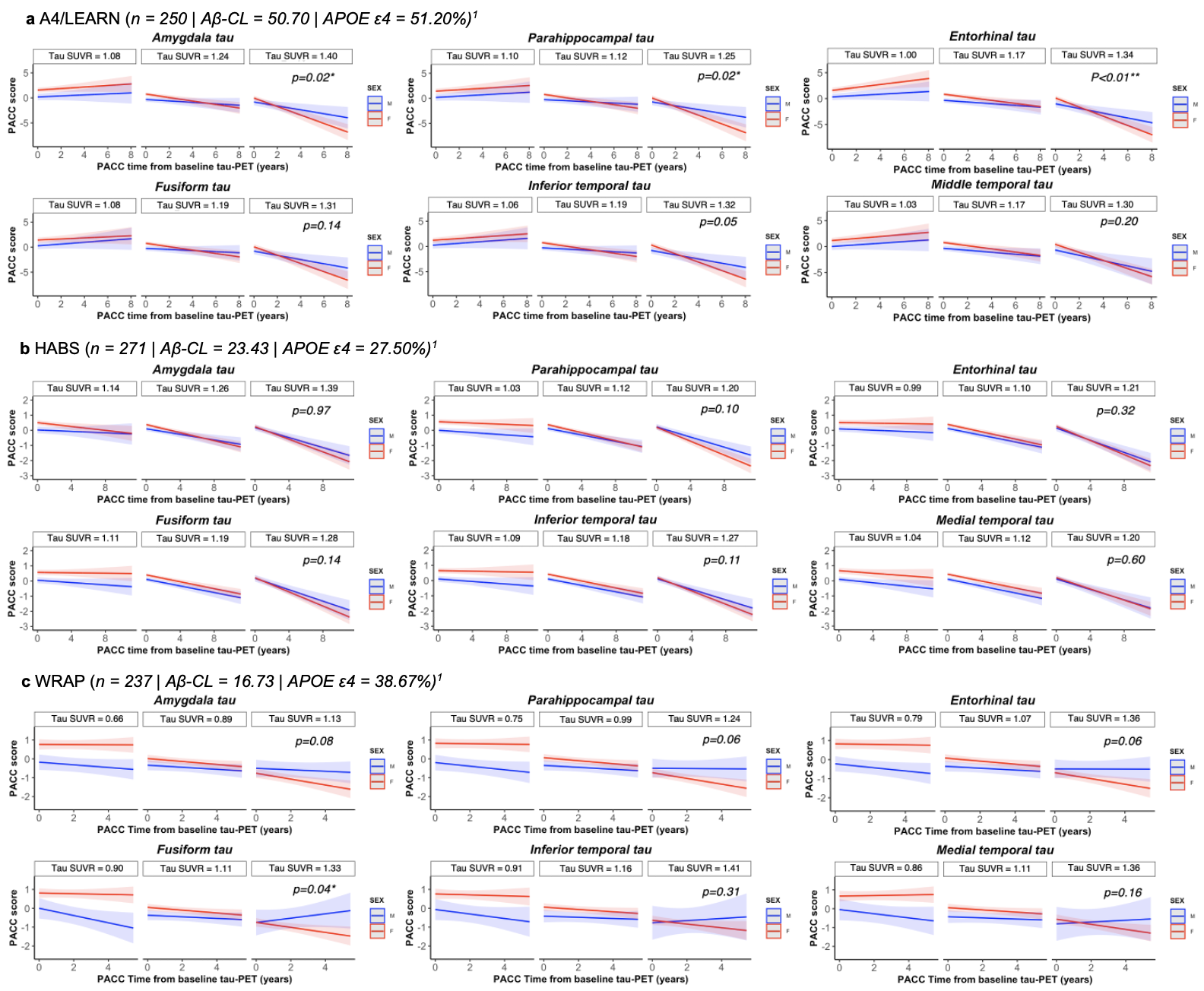


^1^Average Aβ-CL and *APOE*ε4 carrier frequency for each cohort

Three-way linear interaction between sex and regional tau-PET on prospective PACC scores over time, indicating that sex moderates the relationship between medial temporal tau and cognitive decline in A4/LEARN and WRAP. a-c: Cohort-specific interaction visualizations: a: A4/LEARN (N=250), b: HABS (N=271), and c: WRAP (N=237), depicting the association between sex, regional tau-PET, and prospective cognitive trajectories post-tau-PET scan. In all plots, * denotes p<0.05 and ** denotes p<0.01. Cognitive trajectories are faceted by tau SUVR values corresponding to the mean and ±1 standard deviation of regional tau burden within each cohort.
